# Comparing the effect of body wash with marshmallow plant and lukewarm water on reducing the temperature of febrile children: a randomized clinical trial

**DOI:** 10.1186/s12906-022-03762-3

**Published:** 2022-11-12

**Authors:** Hadis Goodarzi, Fatemeh Valizadeh, Fatemeh Ghasemi, Farzad Ebrahimzade, Seyedeh Hanieh Seifosadat, Bahram Delfan, Nadereh Taee

**Affiliations:** 1 Student Research Committee, Lorestan University of Medical Sciences, Khorramabad, Iran; 2 Razi Herbal Medicines Research Center, Department of pediatrics nursing, School of Nursing and Midwifery, , Lorestan University of Medical Sciences, Khorramabad, Iran; 3 Social Determinants of Health Research Center, Department of pediatrics nursing, School of Nursing and Midwifery, Lorestan University of Medical Sciences, Khorramabad, Iran; 4 Nutritional Health Research Center, Lorestan University of Medical Sciences, Khorramabad, Iran; 5 School of Pharmacy, Lorestan University of Medical Sciences, Khorramabad, Iran; 6 Department of physiology and pharmacology, School of Medicine, Razi Herbal Medicines Research Center, Lorestan University of Medical Sciences, Khorramabad, Iran; 7 School of Medicine, Lorestan University of Medical Sciences, Khorramabad, Iran

**Keywords:** Fever, Children, Marshmallow plant, Acetaminophen, Body wash

## Abstract

**Background:**

Fever is the most common reason for children’s visits to medical centers. Its management is an essential duty of a pediatric nurse. The aim of this study was to determine the effect of body wash with Marshmallow plant on children’s fever.

**Methods:**

This parallel clinical trial was performed on 92 children aged 6 months to 10 years with a tympanic temperature above 38.3 °C. Participants were randomly assigned to groups. Simultaneously with receiving acetaminophen, body wash was performed in the control group with lukewarm water and in the intervention group with white Marshmallow extract. The children’s temperature; from the beginning of the study was checked and recorded every 15 min in the first hour and in the 4th and 6th hours. The time duration to resolve fever, the frequency of afebrile children at different times of the study, and the value of temperature reduction were primary outcomes. Heart rate, the need to administer the next dose of acetaminophen, and the time of fever recurrence were recorded as secondary outcomes.

**Results:**

The mean time duration to resolve fever in the intervention group was shorter than in the control group (B = 8.181, 95% CI 3.778–12.584, p < 0.001). The frequency of the children without fever was higher in the intervention group during different times of the study (p < 0.001). The mean value of temperature reduction in the intervention group was higher than the control group (B = -0.27 °C, 95% CI: -0.347 to -0.193, P < 0.001), although, after adjusting the effect of confounding variables it was not significant (P = 0.127). The mean of adjusted heart rate change (p = 0.771), the time of fever recurrence (P = 0.397), and the frequency of children requiring the next dose of acetaminophen (p = 0.397) did not show a significant difference between the groups.

**Conclusion:**

Body wash with Marshmallow extract reduced children’s fever in a shorter period of time and to some extent a greater extent than the control group without side effects. Therefore, it can be used as an effective and safe complementary method to help reduce fever. However, more studies are necessary for this field.

**Trial registration:**

Registration in Iranian Clinical Trials (RCTs) on 31.08.2020 with registration code: IRCT20200809048345N1.

## Background

Fever is one of the most important symptoms of the disease [1] and the most common reason for children’s visits to health centers [1, 2]. The prevalence of children’s visits to a physician due to fever has been reported in some sources to be 19 to 30% [3]. Fever occurs as a result of the reaction to the pyrogenic agents during infection and inflammatory processes [4]. Fever increases body metabolism, anorexia, heart rate, and dehydration. It can lead to sleep disturbances, seizures, brain damage, and death in children [5–7]. Fear of fever, which is a persistent problem among parents and health care providers [8], causes parents to pay frequent visits to health care centers, spend time and money, use inappropriate treatment, and poison children due to the non-standard use of drugs [9]. Fever management is one of the most important duties of pediatric nurses [10, 11]. They manage fever in children in both pharmacological and non-pharmacological methods [10]. Acetaminophen and ibuprofen are two approved drugs for treating fever in children. Non-pharmacological treatments include rest, adequate nutrition, and cooling measures such as body wash and bathing with a sponge, removal of children’s clothes, and oral or intravenous hydration. The body wash is one of the physical and non-pharmacological measures, which is done with a sponge and different solutions such as cold water, alcohol [16, 17], lukewarm water [18], and medicinal plants [1].

Medicinal plants are widely used as complementary and alternative medicines in the treatment of children [19]. Parents often use them for their children, because they believe that herbal remedies are more natural and safer than conventional medications [20]. People avoid chemical drugs due to the fear of side effects and are more interested in using herbal medicines [21, 22]. Various plants can have a positive effect on fever including Viola Odorata L [23], Beetroot, Chicory, Coriander, Leeks, Licorice, Chamomile, Mint, Turmeric, Willow, and Marshmallow [24].

The Marshmallow plant belongs to the Malvaceae family and its scientific name is Althea officinalis [25], which means healing [26]. Marshmallow is native to Asia, Europe, and the United States [27]. In Iran, it is distributed in the northwest to the northeast [28], southwest [29], and west of the country such as Lorestan province [30, 31]. The native name of Marshmallow in Lorestan is Gol Hiro [25]. Marshmallow is a perennial herbaceous plant with broad, toothed leaves. The roots, stems, and leaves of this plant are used in traditional medicine [32]. Marshmallow root contains 91% mucilage compounds, 99% starch, 19% sucrose, betaine, flavonoids, coumarins, oil [33], and polyphenols [34]. It has white, pale pink, and red flowers [27, 35]. The highest amount of flavonoids is in white Marshmallow [36]. Marshmallow is a stimulant of the immune system and has antibiotic and anti-inflammatory properties. It is used in the treatment of oral disorders, respiratory tract and chest diseases, bronchitis and severe cough [37], kidney disorders, gastrointestinal tract diseases, and hypoglycemia. In addition, it has been used in the treatment of eczema, psoriasis, food poisoning, normalization of metabolism [32, 33], headache and migraine [38], wound healing and skin lesions [39], chemotherapy-induced stomatitis [40], common colds [25], atopic dermatitis [41], acute nonbacterial tonsillitis [42] and to reduce the body temperature [43].

Because fever is a common health problem in children and has complications, parents’ intense fear and anxiety often lead them to use substances such as alcohol, salt, and … with body wash by mistake, which in turn carry risks. One of the common methods of body wash used by parents is the use of medicinal plants. Since the Marshmallow is a native, cheap and accessible medicinal plant in Lorestan province, the present study aimed to compare the effect of body wash with lukewarm water and Marshmallow extract on lowering the temperature of children with fever along with the use of acetaminophen.

## Methods

### Study design and settings

This study is a blinded parallel-randomized controlled clinical trial. It was performed after approving the project and obtaining the code of ethics from Lorestan University of Medical Sciences (IR.LUMS.REC.1399.087) and registering in the Iranian randomized clinical trial system (IRCT20200809048345N1). The study was conducted for 6 months from October 2020 to March 2021.

### Study participants and sample size

**Fig. 1:**
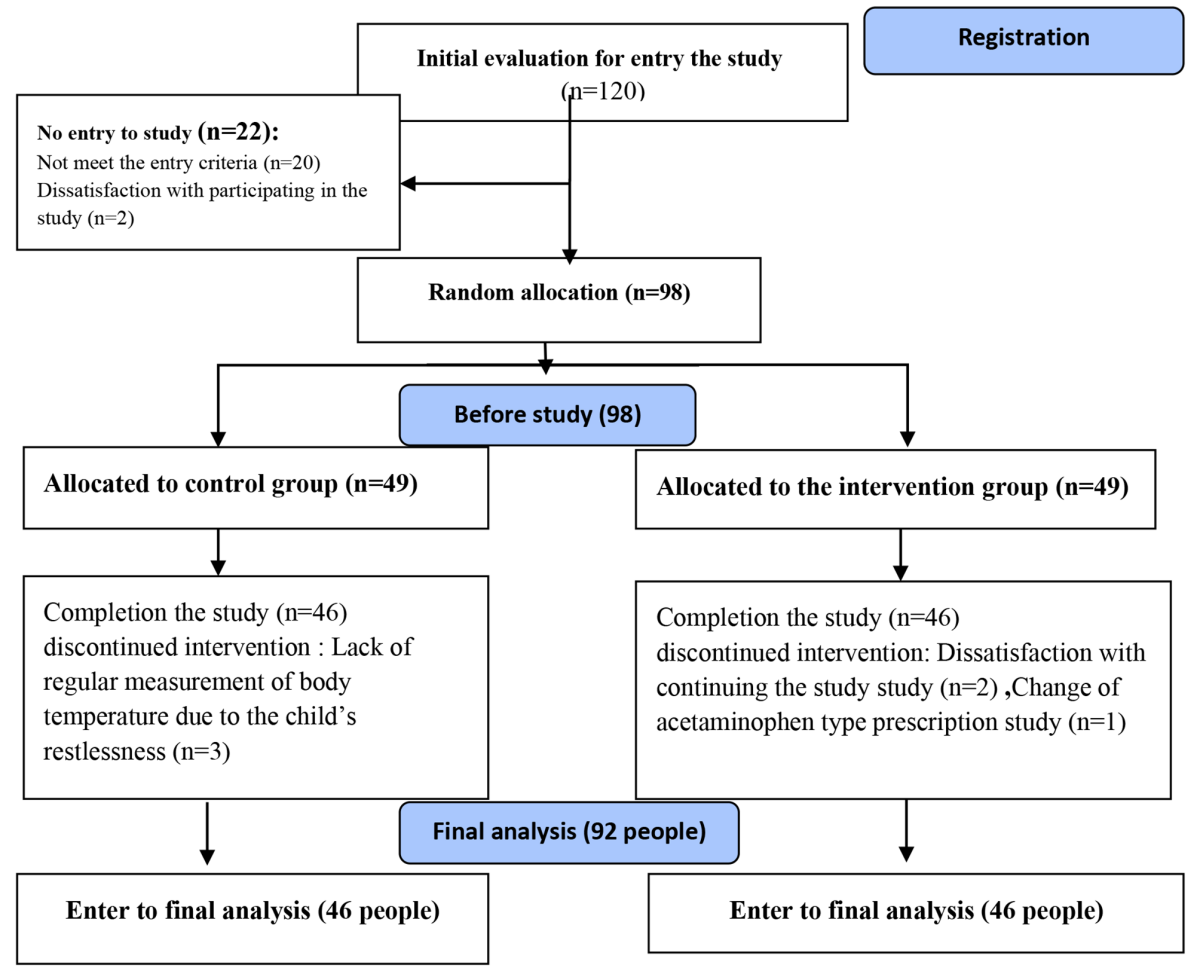
CONSORT flow chart of sampling

Inclusion criteria were; the children’s age of between 6 months and 10 years; having a tympanic temperature of above 38.3 °C, and having a prescription for acetaminophen suppository and body wash in case of fever. Children with a history of febrile seizure, chronic illness, using corticosteroids, allergy to acetaminophen and herbs (Marshmallow) in them or their families, had prescriptions for antipyretic drugs other than acetaminophen and those who were dehydrated were not included in the study. Also, those who showed signs and symptoms of allergies or peripheral circulatory collapse or seizures during the study; had a fever of above 40 °C resistant to treatment, and finally children or parents did not cooperate to continue participating in the study were excluded. The sample size was calculated to be 40 people in each group based on a similar study by Ebadinejad et al. [43], α = 0.05, β = 0.2, CI = 0.95, power = 0.8 and using PASS software version 15, however, after taking into account the probability of 20% sample drop, 49 people were considered for each group (Fig. 1).

### Study arms, randomization and blinding

Participants were enrolled in the study by continuous non-probability sampling method from eligible samples according to the chronological order of hospitalization. The patients were divided into two groups by stratified random sampling. Groups were made based on the diagnosis (gastrointestinal diseases, respiratory diseases, etc.), age (less than 24 months, 24 months and older), and sex (boy and girl) of the children. Within each group, the method of random permutation blocks was used to allocate samples to the intervention and control groups. The block’s lengths were binary and the randomization tool was a random table of numbers. The study was conducted blinded so that, the children’s temperature was measured and recorded by a nurse who was neither part of the research team nor aware of the type of body wash solution. The statistical consultant was also not aware of the groups during the data analysis.

### Study procedures

For all children, a stationary nurse who was not a member of the research team initially measured the temperature with a tympanic thermometer. If children had a tympanic temperature above 38.3° C, one of the researchers would have examined them for other inclusion criteria. Having the criteria to enter the study, the purpose of the study was explained to the children in a suitable language and to their parents. Then, those who consent to participate were randomly assigned to one of the study groups. The statistician identified a random allocation sequence and one of the researchers used that to assign participants to the groups.

### Study intervention

In the present study, the flower of white Marshmallow was used to prepare the extract. A botanist at Razi Herbal Medicines Research Center then identified the plant materials, and a voucher sample (No. 13,971,479) was archived at the herbarium of Razi Herbal Medicines Research Center, Khorramabad, Iran. Preparation of aqueous extract of white Marshmallow was done in the laboratory of Khorramabad School of Pharmacy by observing the principles of extraction by a colleague who was an expert in medicinal chemistry under the supervision of the pharmacist associate of the project. According to previous studies [43], 5 g of ground Marshmallow was mixed in 1 L of distilled water at 20 °C for 8 h. Then, the Whatman filter paper number 41 with a porosity of 20 microns was used to extract the Marshmallow. Then, the extract solution was placed in 1-liter disposable bottles for use in the intervention. The extract was prepared and stored at the appropriate temperature in the treatment room of hospital wards for use. For the control group, ordinary water in the 1-liter disposable bottles was prepared and kept in the the hospital wards at a suitable temperature for daily use the same way as the extract solution.

Simultaneously with the use of acetaminophen suppository (as ordered by the physician), body wash was performed in the control group with lukewarm water, and in the intervention group with white Marshmallow extract. In both groups, one liter of body wash solution was warmed up by Routel ch2823U electric kettle to the temperature of 28.84 ± 1.8 °C. The standard method of body wash was done by placing a cotton cloth (with dimensions of 50 * 50 cm folded four) on the neck, armpits, and groin areas (in a rotation manner and up to two areas at a time). One of the researchers did the intervention. Although she was aware of the type of body wash solution because of the slight smell of the solution, she had no role in measuring the outcomes. The outcomes were measured by a nurse who was not a member of the research team and was not aware of the type of body wash solution and the allocation of children to the groups. The surface temperature of the washing cloth was controlled with a 100 TB digital thermometer at the beginning of the study and every 15 min during the body wash, and as soon as it reached less than 27.04 °C, the washing cloth was replaced with a new one. The temperature of the body wash solution was measured every 15 min during the intervention with an alcohol thermometer, and as soon as it reached less than 27 °C, the solution temperature was reheated to 28.84 ± 1.8 °C by Routel ch2823U electric kettle. Body wash was continued until the children’s temperature returned to less than 38.3 °C.

In all stages, the children’s body temperature was checked and recorded by a tympanic thermometer model 100 TB with an accuracy of 0.1 °C within 30 s. To prevent infection, the tympanic thermometer was disinfected with alcohol cotton every time before measuring the temperature and the disposable cover of the thermometer speculum was changed from one child to another. The children’s heart rate was also checked and recorded by a stethoscope for one minute. Also, the ambient temperature was recorded by a wooden thermometer. These measures were taken at the beginning of the study and then every 15 min until the temperature returned to the normal range as well as at the 4th and 6th hours after the start of the study. During the study, the children were closely monitored for signs of peripheral vascular collapse as well as allergic symptoms.

### Clinical outcome measures

A demographic questionnaire and a researcher-made form were used to collect and record the data. The demographic questionnaire included questions about age, sex, weight, the number of previous hospitalizations, history of febrile seizures in the child and family, level of education of the mother, history of illness or use of certain drugs. It was completed by asking the mothers. A part of the researcher-made form was used to record diagnosis, medication and fluids usage which was completed by studying the child’s hospital file. Another part of the researcher-made form was used to record temperatures of the child, ambient, washing cloth and body wash solution at the start of the study and every 15 min until the return of tympanic temperature to less than 38.3 °C and also at 4th and 6th hours after the start of the study. The mean decrease in children’s temperature and the mean time duration that fever was resolved after the beginning of body wash and the frequency of children without fever in each group during different times of study were the primary outcomes. The time of fever recurrence and the number of acetaminophen doses received during the study, the complications of body wash, including symptoms of peripheral vascular collapse (increased heart rate, chills, and decreased level of consciousness) and allergy symptoms (redness, itching, and any other discomfort during the study) were examined at the onset of intervention, and every 15 min until the elimination of fever, and also at 4th and 6th hours after the start of the study as the secondary outcomes. In case of any problems, they were recorded in the researcher-made form.

### Data analysis

Data analysis was performed by SPSS software version 18 and SAS software version 9.1 at the significance level of 0.05. Kolmogorov-Smirnov test was used to assess the data normality. To describe the data, frequency distribution table, mean and standard deviation, or median and interquartile range were used. For univariate comparisons between the two groups, the Chi-square independence test, Fisher’s exact test, independent t-test, Mann-Whitney test, and Log-rank survival test were used. Washing cloth and solution temperature were compared by joint modeling of survival and longitudinal data and the number of acetaminophen doses received during the study was compared by the generalized linear model (with a Poisson probability distribution and logarithmic link function). Another generalized linear model (with a normal probability distribution and identical link function) compared the first time that temperature returned to the normal range. The fever recurrence after 6 h was compared by Firth’s logistic regression model. A marginal model for longitudinal data compared the child’s body temperature status and heart rate changes at different minutes. To ensure the correct interpretation of study results, the effect of confounding variables was adjusted in the final multivariable model. Those demographic or background variables that had a p-value less than 0.2 in univariate comparisons between two groups, their effect were adjusted in multivariate modeling. These variables were: vancomycin, zinc, meropenem, amount of fluids received during the study, average ambient temperature, average washing cloth temperature, and average solution temperature.

## Results

The study was performed on 92 children who had been allocated to two groups intervention and control (Fig. 1). There was no significant difference between the groups in terms of demographic variables, such as gender, age, diagnosis, number of previous hospitalizations, weight, body temperature at the research initiation, the amount of fluid intake during the study, the time-lapse since the administration of the last dose of acetaminophen, and the education of child’s mother (Table 1).

**Table 1:** Comparison of intervention and control groups in terms of demographic and background characteristics

Variable Group		Intervention	Control	P-value
		N (%) Mean ± SD	N (%) Mean ± SD	
Gender	Girl	22 (47.8)	22 (47.8)	^*^0.999
	Boy	24 (52.2)	24 (52.2)	
Age group	Less than 24 months	(50.0) 23	(50.0) 23	^*^0.999
	24 months or more	(50.0) 23	(50.0) 23	
Disease/ diagnosis	Respiratory	(17.4) 8	(17.4) 8	^*^0.999
	Digestive	(34.8) 16	(34.8) 16	
	Other	(47.8) 22	(47.8) 22	
Mother’s education level	Illiterate	(4.4) 2	(13.0) 6	^*^0.369
	Below high school diploma	22 (47.8)	24 (52.2)	
	High school diploma	14 (30.4)	11 (23.9)	
	University education	8 (17.4)	5 (10.9)	
Number of previous hospitalization	Not admitted	16 (17.4)	17 (18.5)	^*^0.880
	Once	19 (20.7)	20 (21.7)	
	Twice or more	11 (12.0)	9 (9.8)	
Child’s weight before study (Kg)		12.17 ± 3.81	13.05 ± 5.52	^**^0.775
Fluid intake during the study (CC)		826.09 ± 369.04	715.22 ± 433.57	0.078^**^
Time-lapse since receiving the last dose of acetaminophen at the start of study (Min)		365.74 ± 15.78	357.39 ± 12.37	0.992^***^
Temperature at the research initiation		38.80 ± 0.28	38.87 ± 0.29	0.233^****^

*Chi-square **Mann-Whitney test *** Log-rank survival test **** Independent t-test

Fisher’s exact test showed a significant difference between the two groups in terms of using vancomycin (P = 0.012), but in terms of other drugs such as zinc, meropenem, ceftriaxone, clindamycin, probiotics, gentamicin, and others, there was no statistically significant difference between the groups.

The Independent t-test showed no statistically significant difference between the two groups in terms of ambient temperature (P = 0.088). Also, the joint modeling of survival and longitudinal data (random effects model) showed that by adjusting the effect of the time factor, no statistically significant difference was observed between the intervention and control groups in terms of the distribution of washing cloth and solution temperatures ​​​​(Table 2).

**Table 2:** Comparison of intervention and control groups in terms of ambient, washing cloth and solution temperature

Group Variable		Intervention Mean ± SD	Control Mean ± SD	P-value
Ambient temperature	At the start (0 min)	28.04 ± 0.45	27.87 ± 0.52	0.088*
	at 15th minute	28.04 ± 0.45	27.87 ± 0.52	
	at 30th minute	28.04 ± 0.45	27.87 ± 0.52	
	at 45th minute	28.04 ± 0.45	27.87 ± 0.52	
	at 60th minute	28.04 ± 0.45	27.87 ± 0.52	
	at 4th hour	28.04 ± 0.45	27.87 ± 0.52	
	at 6th hour	28.04 ± 0.45	27.87 ± 0.52	
Washing cloth temperature	At the start (0 min)	28.309 ± 0.457	28.561 ± 0.364	0.122**
	at 15th minute	28.998 ± 0.471	28.246 ± 0.335	
	at 30th minute	27.712 ± 0.311	27.959 ± 0.309	
	at 45th minute	27.400 ± 0.357	27.673 ± 0.282	
	at 60th minute	-	27.800 ± 0	
Solution temperature	At the start (0 min)	29.674 ± 0.429	29.609 ± 0.370	0.067**
	at 15th minute	29.235 ± 0.425	29.148 ± 0.338	
	at 30th minute	28.788 ± 0.423	28.723 ± 0.376	
	at 45th minute	28.117 ± 0.560	28.360 ± 0.302	
	at 60th minute	-	27.700 ± 0	

* Independent t-test ** Simultaneous modeling of survival and longitudinal data

### Primary outcomes

The mean time duration that fever was resolved after the beginning of body wash: The mean ± standard deviation of body temperature return to normal value was 26.41 ± 11.48 in the intervention group and 39.78 ± 9.07 in the control group. The generalized linear model (normal probability distribution and the identical link function) showed that, after adjusting the effect of the confounding variables, the effect of the intervention on the meantime of temperature return to the normal range was significant (χ = 13.236, df = 1 and P < 0.001). So that the intervention was able to reduce the time it took for the temperature to return to the normal range by an average of 8.181 min, which was statistically significant (B = 8.181, 95% CI 3.778–12.584: and P < 0.001).

The frequency of the children without fever: During different minutes after the start of the study ( in the 15th, 30th, 45th minutes and 6th hour) the frequency of the children without fever was higher in the intervention group (P < 0.001) (Table 3). Marginal model (method of estimating GEE parameters, binomial probability distribution, logit link function, and structure of exchangeable correlation matrix) after adjusting the effect of confounding variables showed that the interaction effect of the intervention, and time factor on the frequency of children without fever during the study was statistically significant (χ^2^ = 13.299, df = 1 and P < 0.001, OR = 10.863, 95% CI: 3.014–39.149, β = 2.385).

**Table 3:** Comparison the frequency of febrile children between the intervention and control groups at different intervention minutes

Group Body temperature		Intervention N (%)	Control N (%)	P-value (main intervention effect)	P-value (main effect of time)	P-value (interaction effect of intervention and time)
at 15th minute	Normal	27 (58.7)	6 (13.0)	< 0.001	< 0.001	< 0.00﻿1
	Feverish	19 (41.3)	40 (87.0)			
at 30th minute	Normal	39 (84.8)	27 (58.7)			
	Feverish	7 (15.2)	19 (41.3)			
at 45th minute	Normal	46 (100)	45 (87.8)			
	Feverish	0 (0.0)	1 (2.2)			
at 60th minute	Normal	46 (100)	46 (100)			
	Feverish	0 (0.0)	0 (0.0)			
at 4th hour	Normal	46 (100)	46 (100)			
	Feverish	0 (0.0)	0 (0.0)			
at 6th hour	Normal	46 (100)	43 (83.5)			
	Feverish	0 (0.0)	3 (6.5)			

The mean decrease in children’s temperature: Marginal model (normal probability distribution, identity link function, GEE parameter estimation method, and exchangeable covariance matrix structure) showed that the effect of the intervention on the mean change of body temperature was significant (χ^2^ = 47.377, df = 1 and P < 0.001). The intervention reduced the adjusted mean temperature of the patients’ bodies by -1.0714 °C in the group of body wash with Marshmallow and in the control group by -0.8402° C. It means that, the adjusted mean decrease in patient’s body temperature in the intervention group was 0.27 °C higher than the control group (B = -0.270, 95% CI: -0.347 to -0.193 and P < 0.001) (Table 4). However, after adjusting the effect of confounding variables, the interaction effect of the intervention, and time factor on the mean change of patient’s body temperature was not significant (χ^2^ = 2.333, df = 1 and P = 0.127).

**Table 4:** Comparison the intervention and control groups in terms of decrease in the children’s body temperature in different times of the study

Group Body temperature changes	Intervention		Control		P-value (main intervention effect)	P-value (main effect of time factor)	P-value (interaction effect of intervention and time factor)
	Mean	SD	Mean	SD			
at 15th minute	-0.47	0.14	-0.26	0.11	< 0.001	< 0.001	0.127
at 30th minute	-1.13	0.20	-0.87	0.20			
at 45th minute	-0.81	0.23	-0.53	0.20			
at 60th minute	-1.42	0.24	-1.20	0.24			
at 4th hours	-1.64	0.27	-1.50	0.44			
at 6th hours	-1.17	0.26	-1.02	0.41			

### Secondary outcomes

The number of acetaminophen doses received during the study: The generalized linear model (Poisson probability distribution and logarithmic link function) showed that after adjusting the effect of confounding variables for distribution and number of acetaminophen doses received during the study, although the intervention reduced the relative rate of acetaminophen use by 8.4%, this difference was not statistically significant (P = 0.693, OR = 0.916, CI 95%: 0.591–1.419, β = -0.088).

The time of fever recurrence: Firth’s logistic regression model showed that after adjusting the effect of confounding variables, the effect of the intervention on the incidence of fever recurrence was not statistically significant (P = 0.397, OR = 0.101, 95% CI: 0.444–2.544, β = 2.709).

The complications of body wash: about increased heart rate as an indicator of peripheral vascular collapse Marginal model (with normal probability distribution, identity link function, GEE parameter estimation method, and exchangeable covariance matrix structure) showed that after adjusting the effect of confounding variables, the main effect of the time factor on mean changes in patient’s heart rate was statistically significant (χ^2^ = 88.576, df = 1 and P < 0.001). However, the main effect of the intervention (P = 0.771) and the interaction effect of the intervention and time factor on the mean change in patient’s heart rate were not statistically significant (P = 0.415, df = 1, χ^2^ = -0.665). Other symptoms of peripheral circulatory collapses (chills, and decreased level of consciousness) and symptoms of allergies (redness, itching, and any other discomfort) were not observed in any of the groups.

## Discussion

According to the results, the meantime duration of temperature return to the normal range was significantly 8 min shorter in children receiving Marshmallow body wash than in children in the control group. Consistent with this result Ghorbani and colleagues conducted a study on the effect of hydraulic extract of Marshmallow root on chemotherapy-induced stomatitis and showed the mean time of stomatitis clearance was shorter in the intervention group than in the control [40]. In Ebadinejad’s study, the body temperature of most children returned to the normal range within 30 min, but this time in the control group was 45 min and even more [43]. Therefore, it can be concluded that body wash with Marshmallow extract can reduce fever more quickly than lukewarm water. Which is very important from a clinical point of view. Because the standard time interval for controlling the temperature is every 15 min. Therefore, body wash with marshmallow has lowered the fever by almost half of this time interval. Which can sooner reduce the parents’ fever phobia and inappropriate treatment will be less. In addition, this result will shorten the time spent on fever care for nurses.

The relative frequency of children with normal temperature in the group receiving Marshmallow body wash was significantly higher than the control group from the 15th minute until the 6th hour. In line with the findings of the present study in the study of Ebadinejad and colleagues, in the Marshmallow body wash group, the relative frequency of children with normal temperature was higher than in the control group [43]. Jafarimanesh and colleagues reported less relative frequency of people with latex allergy symptoms in the Marshmallow group [44]. In evaluating the effectiveness and safety of anti-cough granules containing Marshmallows, Ivy, and Sorrel plants, Khan and colleagues found that more people improved in the intervention group [45]. Thus, it can be concluded that Marshmallow extract, which is absorbed through the skin and is effective on the symptoms of skin inflammation, possibly also be absorbed through the skin when used as body wash and reduce fever as a systemic sign of inflammation.

The mean decrease in body temperature of children Marshmallow body wash was 0.27 °C higher than the control group. The study by Ebadinejad and colleagues showed that the rate of decrease in body temperature of children in the Marshmallow body wash group was higher than in the lukewarm water body wash group [43]. Although this effect was not significant in the present study after adjusting for confounding variables, it is clinically valuable. Because clinically, the temperature change can be checked at 0.1 °C. Therefore, marshmallow has reduced the fever by more than two and a half times the minimum significant temperature difference, more than body wash with lukewarm water. It shows that Marshmallow can reduce the consumption of anti-fever drugs as a complementary treatment, as the amount of fever drug consumption was decreased by 8.4% in the study. Also, in the present study, a small amount of Marshmallow was used for the body wash. Repeating the study with larger amounts of Marshmallow may lead to even better results.

The relative frequency of children requiring the next dose of acetaminophen 4 h after the study in the control group was not different from the group of Marshmallow body wash. However, 6 h after the study, it was higher in the control group than the intervention group, although this difference was not statistically significant. According to a study by Kouchakzadeh Talemi et al., the use of intermittent body wash along with acetaminophen was effective in reducing the body temperature of children with fever at 4 h after the intervention [5]. Zeighami and colleagues’ study showed that footbath intervention could reduce patients’ body temperature within 4 h [46]. In Tan Unicia and colleagues’ study acetaminophen reduced fever in less than 4 h [47]. These studies are similar to the control group in the present study. However, in the Marhmallow body wash, this effect was prolonged up to 6 h, although this difference was not significant. The reason for this may be using a low dose of Marshmallow extract in the present study. Perhaps, using a higher dose of Marshmallow can confirm its effectiveness in this regard.

The study found that none of the children showed symptoms of allergy and peripheral vascular collapse following body wash. This means that while Marshmallow extract returned the temperature to normal values ​​in a shorter period and greater extent, it did not have any side effects. Therefore, possibly it can be an effective and safe method to reduce children’s fever. Studies have shown that the white Marshmallow plant has the highest level of flavonoids and can be useful for medicinal purposes [36]. Polysaccharide is another compound in Marshmallow that has a stimulating effect on the immune system [48]. The aqueous extract of Marshmallow stimulates phagocytosis to release oxygen radicals and leukotrienes from neutrophils. Marshmallow also has anti-inflammatory and antioxidant effects due to the presence of alpha-tocopherol and increased mucin secretion [49]. The active ingredient of Marshmallow, especially mucilage, is effective in inhibiting inflammation by inhibiting tumor necrosis factors [50], cyclooxygenase and prostaglandin production, and by causing sweat and subsequent evaporation, it reduces body temperature in a febrile patient. Since fever is one of the symptoms of inflammation, Marshmallow can be effective in reducing fever with its antipyretic properties (even in topical use) [35]. This plant is effective not only in improving infectious diseases but also in other febrile diseases by strengthening the immune system [51, 52].

The present study showed that the joint use of medicinal and herbal methods, without causing side effects, leads to faster control of children’s body temperature. This method had many advantages such as helping to prevent the side effects of fever in children and reducing parental stress and discomfort. This can make the child more relaxed, reduce parental stress and decrease the use of antipyretic drugs to prevent overuse of antipyretic drugs such as acetaminophen and ibuprofen and prevent medicinal poisoning. The Marshmallow plant can be used simultaneously with physical methods to reduce fever [8]. The World Health Organization (WHO) has also suggested the use of physical methods to reduce fever [53, 54]. Many mothers start self-medication at home with a fever before visiting a physician. The most commonly used method to reduce fever by mothers is body wash. This paper provides mothers with a scientifically evaluated method in terms of effectiveness, safety, and side effects. However, the results cannot be generalized to all children, including children aged less than 6 months and older than 10 years, having a history of seizures and fever above 40 °C, which were not investigated in this study.

## Conclusion

Body wash with Marshmallow extract in a shorter time returned the temperature of more children to the normal range compared to the control group. In the intervention group, the return time of temperature to the normal range was on average 8.181 min shorter and the frequency of children with fever from the 15th minute onwards was significantly lower than in the control group. Moreover, no side effects such as peripheral circulatory collapse or allergy signs were observed when using Marshmallow extract. Also, the marshmallow extract compare to the control group reduced the mean temperature more by 0.27 °C, decreased the relative use of the antipyretic drug (acetaminophen) by 8.4%, and decreased the risk of recurrence of fever, although these effects were not statistically significant. Therefore, it can be concluded that Marshmallow extract can be used as an effective and safe complementary method to help reduce fever. Of course, more studies are necessary to confirm these effects.

## Data Availability

If someone wants to request, the data from this study should contact Dr. Fatemeh Valizadeh and Ms. Hadith Goodarzi, who the data set used during the study is kept by them.

## References

[1] Leocadio MC, Jabail AC, Rull JA, Sanchez LA, Sauler RG, Tan AM, Tapispisan JN. Pagdikta (The dictation): the meanings in Filipino mothers’ experience of using herbal plants in the management of their children’s fever. International Journal of Public Health Research. 2011:169–79.

[2] Holper DCA. Fever. Enemy or Friend?: A comparison of the perception and management of childhood fever between parents in Germany, Luxembourg and the Netherlands. - Bonn, 2011. - Dissertation, Rheinische Friedrich-Wilhelms-Universität Bonn. Online-Ausgabe in bonndoc: https://nbn-resolving.org/urn:nbn:de:hbz:5 N-25313.

[3] Finkelstein JA, Christiansen CL, Platt R (2000). Fever in pediatric primary care: occurrence, management, and outcomes. Pediatrics.

[4] Anne McIntyre F. Herbal treatment of children: Western and Ayurvedic perspectives. 1st ed., Butterworth-Heinemann Health Sciences; 2005.

[5] Carson SM (2003). Alternating acetaminophen and ibuprofen in the febrile child: examination of the evidence regarding efficacy and safety. Pediatr Nurs.

[6] Fields E, Chard J, Murphy MS, Richardson M (2013). Assessment and initial management of feverish illness in children younger than 5 years: summary of updated NICE guidance. BMJ.

[7] Altun İ, Cinar ND, Walsh A (2011). Psychometric properties of the parents’ fever management scale in a Turkish population. HealthMED.

[8] Crocetti M, Moghbeli N, Serwint J (2001). Fever phobia revisited: have parental misconceptions about fever changed in 20 years?. Pediatrics.

[9] Ansari A, Ravanipour M, Jahanpour F, Hosseini S (2015). The challenges of managing childhood fever by parents referred to health centers in Bushehr. ISMJ.

[10] de Bont EG, Peetoom KK, Moser A, Francis NA, Dinant G-J, Cals JW (2015). Childhood fever: a qualitative study on GPs’ experiences during out-of-hours care. Fam Pract.

[11] Jeong YS, Kim JS (2010). Fever and fever management in children: A literature review. Child Health Nursing Research.

[12] Fields E, Chard J, Murphy MS, Richardson M (2013). Assessment and initial management of feverish illness in children younger than 5 years: summary of updated NICE guidance. BMJ.

[13] Sullivan JE, Farrar HC (2011). Section on Clinical Pharmacology and Therapeutics, Committee on Drugs. Fever and antipyretic use in children. Pediatrics.

[14] National Collaborating Centre for Women’s and Children’s Health (UK. Feverish illness in children: assessment and initial management in children younger than 5 years. 2013. https://pubmed.ncbi.nlm.nih.gov/25340238/. 25340238

[15] Watts R, Robertson J (2012). Non-pharmacological management of fever in otherwise healthy children. JBI Evid Synthesis.

[16] El-Radhi AS (2008). Why is the evidence not affecting the practice of fever management?. Arch Dis Child.

[17] Greisman LA, Mackowiak PA (2002). Fever: beneficial and detrimental effects of antipyretics. Curr Opin Infect Dis.

[18] Eisenman SW, Zaurov DE, Struwe L, editors. Medicinal plants of central Asia: Uzbekistan and Kyrgyzstan. Springer Science & Business Media; 2012.

[19] Simpson N, Pearce A, Finlay F, Lenton S (1998). The use of complementary medicine in paediatric outpatient clinics. Ambul Child Health.

[20] Pitetti R, Singh S, Hornyak D, Garcia SE, Herr S (2001). Complementary and alternative medicine use in children. Pediatr Emerg Care.

[21] Mosihuzzaman M (2012). Herbal medicine in healthcare-an overview. Nat Prod Commun.

[22] Ekor M (2014). The growing use of herbal medicines: issues relating to adverse reactions and challenges in monitoring safety. Front Pharmacol.

[23] Tafazoli V, Shahriari M, Heydari M, Nikbakht HA, Zarshenaas MM, Nimrouzi M (2020). The Effect of Viola Odorata L. Oil for Fever in Children: A Randomized Triple-blinded Placebo-controlled Clinical Trial. Curr Drug Discov Technol.

[24] Hadian F, Varshochi M, Zargaran A, Besharat M, Mousavi Bazaz M (2019). Medicinal Herbs Useful in Pediatric Fever from the Perspective of Persian Medicine. Int J Pediatr.

[25] Delfan B, Kazemeini H, Bahmani M (2015). Identifying effective medicinal plants for cold in Lorestan province, West of Iran. J evidence-based Complement Altern Med.

[26] Austin DF, Honychurch P. In: Florida Ethnobotany: CRC press.2004, p. 57.

[27] Ali Shah SM, Akhtar N, Akram M, Akhtar Shah P, Saeed T, Ahmed K, Asif HM (2011). Pharmacological activity of Althaea officinalis L. J Med Plants Res.

[28] Valiei M, Shafaghat A, Salimi F (2011). Chemical composition and antimicrobial activity of the flower and root hexane extracts of Althaea officinalis in Northwest Iran. J Med Plants Res.

[29] Mardaninejad S, Janghorban M, Vazirpour M (2013). Collection and identification of medicinal plants used by the indigenous people of Mobarakeh (Isfahan), southwestern Iran. J Herb Drugs (An Int J Med Herbs).

[30] Bahmani M, Rafieian-Kopaei M, Avijgan M, Hosseini S, Golshahi H, Eftekhari Z (2012). Ethnobotanical studies of medicinal plants used by Kurdish owner’s in south range of Ilam province, west of Iran. Am-Euras J Agric Environ Sci.

[31] Jivad N, Bahmani M, Asadi-Samani M (2016). A review of the most important medicinal plants effective on wound healing on ethnobotany evidence of Iran. Der Pharmacia Lettre.

[32] Kianitalaei A, Feyzabadi Z, Hamedi S, Qaraaty M (2019). Althaea Officinalis in Traditional Medicine and modern phytotherapy. J Adv Pharm Educ Res.

[33] Hammond N, Boyle M (2011). Pharmacological versus non-pharmacological antipyretic treatments in febrile critically ill adult patients: a systematic review and meta-analysis. Australian Crit Care.

[34] Ciobanu M, Pirvu L, Paun G, Savin S, Albu B-G, Munteanu C (2019). Development of a new (bio) hybrid matrix based on Althaea officinalis and Betonica officinalis extracts loaded into mesoporous silica nanoparticles for bioactive compounds with therapeutic applications. J Drug Deliv Sci Technol.

[35] Al-Snafi AE (2013). The pharmaceutical importance of Althaea officinalis and Althaea rosea: A review. Int J Pharm Tech Res.

[36] Sadighara P, Gharibi S, Jafari A, Khaniki G, Salari S (2012). The antioxidant and Flavonoids contents of Althaea officinalis L. flowers based on their color. Avicenna J phytomedicine.

[37] Mahboubi M (2020). Marsh Mallow (Althaea officinalis L.) and Its Potency in the Treatment of Cough. Complement Med Res.

[38] Delfan B, Bahmani M, Hassanzadazar H, Saki K, Rafieian-Kopaei M (2014). Identification of medicinal plants affecting on headaches and migraines in Lorestan Province, West of Iran. Asian Pac J Trop Med.

[39] Delfan B, Bahmani M, Eftekhari Z, Jelodari M, Saki K, Mohammadi T (2014). Effective herbs on the wound and skin disorders: a ethnobotanical study in Lorestan province, west of Iran. Asian Pac J Trop Disease.

[40] Ghorbani M, Taghadosi M, Akbari H, Sharifi M (2019). Effect of hydroalcoholic extract of Althaea officinalis root on improving chemotherapy-induced stomatitis: A randomized, double-blind, clinical trial. Nurs Midwifery Stud.

[41] Naseri V, Chavoshzadeh Z, Mizani A, Daneshfard B, Ghaffari F, Abbas-Mohammadi M (2021). Effect of topical marsh mallow (Althaea officinalis) on atopic dermatitis in children: A pilot double‐blind active‐controlled clinical trial of an in‐silico‐analyzed phytomedicine. Phytother Res.

[42] Popovych V, Koshel I, Malofiichuk A, Pyletska L, Semeniuk A, Filippova O (2019). A randomized, open-label, multicenter, comparative study of therapeutic efficacy, safety and tolerability of BNO 1030 extract, containing marsh mallow root, chamomile flowers, horsetail herb, walnut leaves, yarrow herb, oak bark, dandelion herb in the treatment of acute non-bacterial tonsillitis in children aged 6 to 18 years. Am J Otolaryngol.

[43] Ebadinejad Z, Dashtgard A, Mohseni Zade M (2017). The effect of Wet spong with Luke warm water and marsh mallow on reducing body temperature of children admitted to the Teaching Hospital-Shohada Qaen. Iran J Pediatr Nurs.

[44] Jafarimanesh H (2015). The effect of Alcea Althea on latex allergy among operating room staffs in Arak Hospitals, Iran. Complement Med J.

[45] Khan M, Akram M, Akhter N, Mukhtiar M, Zahid R, Khan F (2018). The evaluation of efficacy and safety of Cough (EMA) granules used for upper respiratory disorders. Pak J Pharm Sci.

[46] Zeighami R, Haghi M, Bijani B, Alipour M, Kaboudi B, Haghi M (2015). A comparison between cold water sponging and fanning in reducing fever in intensive care unit inpatients: a factorial design. Iran J Nurs Res.

[47] Tan E, Braithwaite I, McKinlay CJD, Dalziel SR (2020). Comparison of acetaminophen (paracetamol) with ibuprofen for treatment of fever or pain in children younger than 2 years: A systematic review and meta-analysis. JAMA Netw open.

[48] Ilina T, Skowrońska W, Kashpur N, Granica S, Bazylko A, Kovalyova A, Goryacha O, Koshovyi O (2020). Immunomodulatory activity and phytochemical profile of infusions from Cleavers herb. Molecules.

[49] Bonaterra GA, Bronischewski K, Hunold P, Schwarzbach H, Heinrich EU, Fink C, Aziz-Kalbhenn H, Müller J, Kinscherf R. Anti-inflammatory and Anti-oxidative Effects of Phytohustil® and Root Extract of Althaea officinalis L. on Macrophages in vitro. Frontiers in pharmacology. 2020, 17;11:290. 10.3389/fphar.2020.00290 PMC7090173 32256361

[50] Stockley JH, Evans K, Matthey M, Volbracht K, Agathou S, Mukanowa J, Burrone J, Káradóttir RT. Surpassing light-induced cell damage in vitro with novel cell culture media. Scientific Reports. 2017. 10.1038/s41598-017-00829-x 10.1038/s41598-017-00829-x PMC5429800 28405003

[51] Organization WH. WHO monographs on medicinal plants commonly used in the Newly Independent States (NIS): Libros Digitales-World Health Organization (WHO); 2010. http://repositorio.ub.edu.ar/handle/123456789/5157.

[52] Avicenna H (2005). The canon of medicine (Al-Qanon fi al-Tibb).

[53] Demir F, Sekreter O (2012). Knowledge, attitudes and misconceptions of primary care physicians regarding fever in children: a cross sectional study. Ital J Pediatr.

[54] Meremikwu MM, Oyo-Ita A (2002). Paracetamol versus placebo or physical methods for treating fever in children. Cochrane Database of Systematic Reviews.

